# Ciliary/Flagellar Protein Ubiquitination

**DOI:** 10.3390/cells4030474

**Published:** 2015-09-02

**Authors:** Huan Long, Qiyu Wang, Kaiyao Huang

**Affiliations:** Key Laboratory of Algal Biology, Institute of Hydrobiology, Chinese Academy of Sciences, Wuhan 430072, China; E-Mails: huanlong@ihb.ac.cn (H.L.); ihbwqy2015@gmail.com (Q.W.)

**Keywords:** flagella, ubiquitination, post-translational modifications, sperm, primary cilia

## Abstract

Cilia/flagella are conserved eukaryotic organelles that play an important role in the control of cell motility and detection of environmental cues. However, the molecular mechanisms underlying ciliary/flagellar assembly, maintenance, disassembly, and signal transduction are not yet completely understood. Recent studies demonstrated that post-translational modifications (PTMs) such as phosphorylation, methylation, glutamylation, and ubiquitination are involved in these processes. In this mini review, we present a summary of research progress in ciliary/flagellar protein ubiquitination, including the ubiquitin conjugation system identified by proteomics as well as the role of ciliary/flagellar protein ubiquitination in flagellar disassembly, motility, and signal transduction. Moreover, we described putative further research directions in the study of ciliary/flagellar protein ubiquitination.

## 1. Introduction

**Figure 1:**
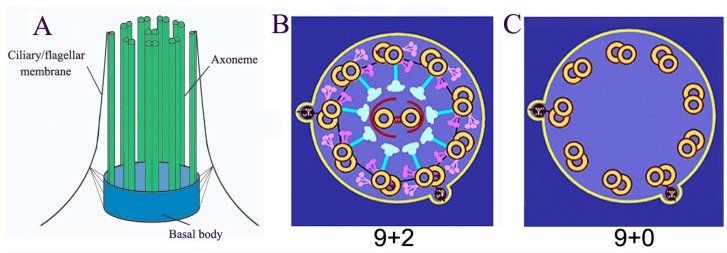
Basal structure of cilia/flagella. (**A**) Cilia/flagella are composed of axoneme, ciliary/flagellar membrane and basal body; (**B**) “9 + 2” motile cilia/flagella possess a central pair and 9 triple microtubule with associated protein complexes such as outer dynein, inner dynein, and radial spoke; (**C**) “9 + 0” immotile cilia/flagella only have 9 triple microtubule.

Cilia/flagella are microtubule-based organelles projecting from the surface of most eukaryotic cells (cilia and flagella are used interchangeably, this review only refers to eukaryotic flagella, not bacterial and archaeal flagella). Cilia are composed of basal body, axoneme (nine triplets of microtubules as the core structure), and the ciliary membrane (Figure 1A). Most motile cilia possess a 9 + 2 structure, in which the axoneme possesses a central pair of microtubules (Figure 1B). However, immotile primary cilia have a 9 + 0 structure (Figure 1C). The main functions of cilia include controlling cell motility, sensing environmental cues, and mediating ciliary signal transduction [1,2,3,4,5]. These functions depend on the homeostasis of the length and the composition of cilia/flagella [1,2,3,4,5]. Ciliary/flagellar length is the result of a balance between assembly and disassembly (turnover) of microtubules at the tip. Since no protein synthesis occurs within the cilia/flagella, all axonemal precursors and turnover products need to be transported via Intraflagellar Transport (IFT), which is critical not only for flagellar assembly and disassembly but also for mediating signal transduction in cilia. Mutations in the IFT system and other conserved ciliary proteins directly result in ciliopathies, such as Polycystic Kidney Disease, repeated chest infections, congenital heart disease, male infertility, blindness, and obesity [6,7,8,9,10]. 

Cilia/flagella are polarized organelles and their assembly/disassembly is highly regulated. Flagella assemble when cells exit from the cell cycle and enter into the stationary phase (G0), while they disassemble prior to the mitosis phase of the cell cycle. The processes of ciliary assembly and disassembly are complex, involving processes such as gene expression, protein transport, and vesicle trafficking. One of the key steps is selective transport. Anterograde IFT interacts with axonemal precursors at the base and transports them to the tips of the cilia, whereas retrograde IFT loads turnover products at the tip and transport them to the base [5,11]. Recent studies demonstrated that various types of PTMs (post-translational modifications) such as phosphorylation, methylation, glutamylation, and ubiquitination are involved in these highly regulated processes. For instance, microtubules undergo acetylation, glycylation, glutamylation, and ubiquitination [12,13,14,15].

The functions of protein phosphorylation in regulating flagellar assembly, disassembly, and length control are well documented [16]. Flagellar assembly requires glycogen synthase kinase (GSK)-3 activity and inhibition of GSK-3β results in short flagella [17]. Phosphorylation of *Chlamydomonas* aurora-like kinase (CALK) is a marker of flagellar length during both assembly and disassembly [18]. The flagellar-shortening mutant FLS1 encodes a cyclin-dependent kinase-like kinase, which regulates the phosphorylation of CALK and CrKinesin13; both these kinases are required for disassembly of the distal portion of *Chlamydomona*s flagella [19]. In addition, one specific proteomic study reported that 89 different phosphoproteins have been identified in the process of flagella shortening, indicating dramatic changes in protein phosphorylation during flagellar disassembly [20].

Methylation is another form of PTM involved in flagellar assembly and disassembly. Schneider *et al.*, first showed that flagella contain a cobalamin-independent form of methionine synthase (MetE), whose amount is lowest in full-length flagella, increases in assembling flagella, and is highest in disassembling flagella [21]. In addition, glutamylation may also play a role in flagellar motility. Tubulin can be polyglutamylated by the tubulin tyrosine ligase like protein (TTLL) family, and tubulin glutamylation is essential for regulation of beating asymmetry [22].

To date, ubiquitin has been found only in eukaryotic organisms. Ubiquitination is responsible for controlling an extensive range of physiological processes such as appropriate cell cycle progression, transcriptional regulation, protein quality control, signal transduction, membrane protein endocytosis, intracellular trafficking, and DNA repair [23]. Ubiquitination is a form of PTMs in which ubiquitin molecules are covalently attached to target proteins by a series of enzymes including ubiquitin-activating enzyme (E1), ubiquitin conjugating enzyme (E2), and ubiquitin ligase (E3) [23,24]. Polyubiquitin chains are generated via an isopeptide bond formed between a carboxyl-terminal glycine of one ubiquitin (G76) and a specific lysine residue of another ubiquitin (*i.e.*, K6, K11, K27, K29, K33, K48, or K63). K48-linked ubiquitination generally functions as a signal for degradation in the 26S proteasome, whereas K63-linked ubiquitination has non-proteolytic functions such as membrane protein trafficking, endocytosis, and DNA repair [23,25]. Monoubiquitin and the K63 chain can be removed from the targets by deubiquitylating enzymes, making these modifications reversible [23].

The majority of proteins with K48-linked ubiquitin chains are degraded by the ubiquitin-proteasome system. When a substrate is coupled with this polyubiquitin chain, it binds to the 26S proteasome complex, in which the substrate is unfolded, cleaved, and degraded into small peptides and then the ubiquitin is recycled [23,26]. 

Recently, protein ubiquitination was found in different cilia/flagella such as flagella of *Chlamydomonas*, sperm flagella and primary cilia. Ubiquitination is involved in regulation of ciliary assembly and disassembly, signal transduction, spermatogenesis, or ciliogenesis. In this mini-review, we focus on ciliary/flagellar protein ubiquitination and discuss its putative functions.

## 2. Ubiquitin Conjugating System is Associated with Cilia

The protein of ubiquitin conjugating system was first found in the flagellar proteomes of *Chlamydomonas* [27], which is an excellent organism to study flagella. It has well-established genetics. The flagella of *Chlamydomonas* are easily isolated, and the genome sequence and proteomic data from both the flagella and basal body are available [27,28]. The *Chlamydomonas* flagellar proteome includes a single E1-activating enzyme, four E2-conjugating enzymes, and three E3-ligases [27,28,29]. In addition, the ubiquitin conjugating system proteins were also found in proteomes of primary cilia and mouse photoreceptor sensory cilium [30,31]. For instance, ubiquitin, ubiquitin-like protein, a single E1 activating enzyme, four E2 conjugating enzymes, and two E3 ligases had been identified in the proteome of the mouse photoreceptor sensory cilium [30]. Thus, the ubiquitin conjugation system is associated with motile cilia and primary cilia.

Direct evidence of the association of ubiquitin conjugation system with flagella was obtained from an *in vitro* flagellar ubiquitination experiment [15]. Human influenza hemagglutinin (HA) labeled ubiquitin was added to isolated flagella, and in the presence of ATP, HA-labeled ubiquitin was conjugated with several flagellar proteins. This result demonstrated that not only the all three types of enzymes of the ubiquitin-conjugation system located in flagella but also they can function together to add ubiquitins to flagellar proteins.

## 3. Flagellar Protein Ubiquitination and Flagellar Disassembly

**Figure 2:**
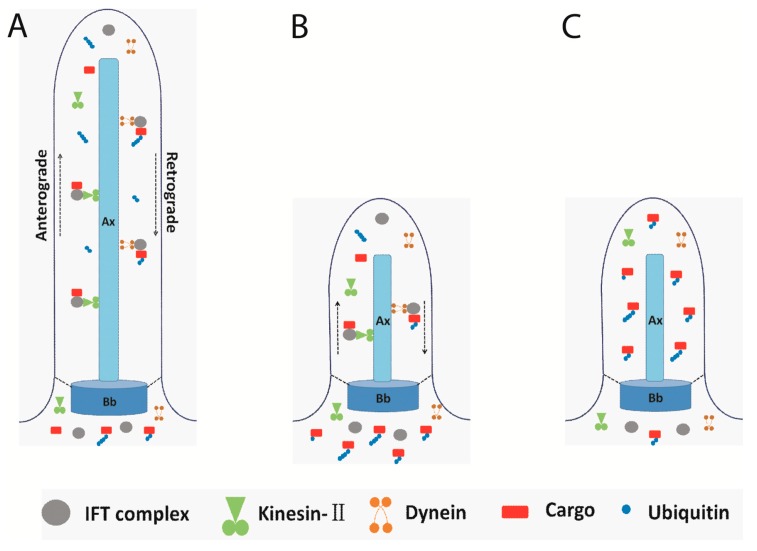
A working model of IFT transporting the ubiquitinated proteins. (**A**) IFT complex moves bi-directionally underneath the flagellar membrane; (**B**) During flagellar disassembly, the turnover products were ubiquitinated and transported to the cell body by retrograde IFT; (**C**) When IFT stops or in retrograde IFT mutants, the ubiquitinated proteins accumulated in the flagella. Ax: Axoneme, Bb: Basal body, dash line indicated transition fiber, solid line indicated flagellar membrane; arrowheads indicated the move direction of anterograde IFT and retrograde IFT.

The activity of ubiquitin conjugation system can also be determined using the *in vitro* flagellar conjugation system. The ubiquitin conjugation activity in flagella was higher when using shortening flagella compared to static flagella, which suggested that ubiquitination is related to flagellar disassembly [15]. Indeed, the pattern of ubiquitinated proteins is similar in assembling flagella as compared to static flagella; however, flagellar ubiquitinated proteins increased during chemically induced flagellar disassembly or naturally occurring flagellar resorption [15]. Furthermore, more ubiquitinated proteins were accumulated in flagella when IFT is blocked, especially in flagella from mutants with defects in retrograde IFT. Since no subunit of the proteasome was found in the *Chlamydomonas* flagellar proteome [27], according to this result, ubiquitinated proteins need to be transported to the cell body. Hence, a working model of the ubiquitin-conjugation system in flagellar disassembly was proposed (Figure 2). The turnover products were labeled with ubiquitin and ubiquitinated proteins were transported to the cell body via retrograde IFT. During flagella shortening induced by NaPPi in wild-type cells, ubiquitinated proteins are transported to the cell body via IFT; therefore, small amounts of ubiquitinated proteins were detected in the shortening flagella. When IFT was blocked in *fla10* at a restrictive temperature or in retrograde IFT mutants, the ubiquitinated proteins could not be transported to the cell body and a large amount of ubiquitinated proteins accumulated in flagella [15]. However, the detailed mechanism needs to be clarified by further studies.

## 4. Flagellar Protein Ubiquitination and Signal Transduction

The ubiquitination may also play a role in regulating signal transduction in flagella. The cation channel CrPKD_2_ and kinase CrPKG, both of which are components of a cAMP-dependent signal transduction pathway in mating, can be ubiquitinated in flagella [15]. The amount of the ubiquitin-conjugating enzyme CrUbc13 also increased rapidly in the flagella after mating and some flagellar proteins were ubiquitinated within 5 min of mixing of the plus and minus gametes [15]. These data suggested that the ubiquitin-conjugating system is not only involved in the regulation of flagellar length but might also participate in regulating flagellar signal transduction [15].

## 5. Flagellar Protein Ubiquitination and Spermatogenesis

Spermatogenesis is the process by which spermatogenic stem cells undergo mitotic and meiotic division and differentiate to streamlined spermatozoa which are capable of motility and fertilization [32]. One of the key events in spermatogenesis is the formation of the flagella, which enables sperm to reach eggs for fertilization. The axoneme, consisting of a conserved central microtubule pair and nine outer microtubule doublets, is the core structure of sperm flagella, and is assembled soon after meiosis [32]. The sperm axoneme is extensively post-translationally modified, including acetylation, tyrosination, and ubiquitination [33,34,35].

The 26S proteasome complex mediating the degradation of polyubiquitinated proteins was isolated from the rat testis and sperm tail in 2000; this finding linked ubiquitination with spermatogenesis [36]. Subsequent work confirmed that the ubiquitin-proteasome system plays a crucial role in spermatogenesis. For example, the RNF8 E3 enzyme mediates histone ubiquitination, which facilitates histone replacement in elongating spermatids [37]. In mammals, the membrane-associated, Really Interesting New Gene (RING)-CH (MARCH) family comprises 11 RING finger-containing E3 ubiquitin ligases. Of these, MARCH7, MARCH10, and MARCH11 are highly expressed in developing spermatids. MARCH7 is localized to the acroplaxome and flagella and may play a role in the regulation of head shaping and flagellar formation [38]. MARCH10 is localized in the fibrous sheath within the principal piece of the flagella and may be involved in flagellar assembly and maintenance in elongating spermatids [39]. MARCH11 may regulate the transport of transmembrane glycoproteins between the trans-Golgi network and multivesicular bodies in round spermatids [40,41]. In addition, aberrant head morphology has been observed in spermatozoa of mice lacking the UBE2B E2 enzyme [42]. Male mice lacking the TATA element modulatory factor/androgen receptor co-activator 160 (TMF/ARA160) E3 enzyme are sterile due to abnormal sperm development such as acrosome disruption and coiled flagella [43,44]. These data suggested that ubiquitination plays a critical role in spermatogenesis.

## 6. Ciliary Protein Ubiquitination in Primary Cilia

The primary cilium functions as an antenna of the cell, protruding directly from the distal end of mother centriole (basal body). The ubiquitin-proteasome system is necessary to initiate ciliogenesis by removing the keratin-binding protein trichoplein from the mother centrioles. Trichoplein is polyubiquitinated at Lys-50 and Lys-57 by Cul3-RING ubiquitin ligase. Degradation of polyubiquitinated trichoplein plays an essential role in the initial step of axonemal extension during ciliogenesis [45]. The E3 ubiquitin ligase Mindbomb 1 (MIB1) is a new component of centriolar satellites; it ubiquitinates Cep131 (also known as AZI1) and pericentriolar material 1 (PCM1) and suppresses primary cilium formation [46]. Taken together, ubiquitination occurs during both initiation and elongation stages of ciliogenesis.

Ubiquitination can function antagonistically or synergistically with other PTMs. For example, tumor suppressor Von Hippel-Lindau protein (VHL) is also an E3 ligase, which stabilizes microtubules in the primary cilium. The kinase Nek1 phosphorylates VHL at multiple sites and promotes its ubiquitination and degradation in the proteasome. Mutation of VHL at serine-168, a Nek1-phosphorylation site, increases the stability of VHL and improves its capacity to stabilize cilia [47]. Apart from protein ubiquitination, protein deubiquitination also plays a role in ciliogenesis. Cylindromatosis tumor suppressor gene (*CYLD*) encodes a deubiquitinating enzyme that removes Lys63- or linear-linked ubiquitin chains, and *CYLD* mutation results in defects of cilium formation [48]. Ubiquitin-specific protease (USP)-8 functions as a deubiquitinating enzyme of Hypoxia-Inducible Factor-1α (HIF1α) by counteracting the Hippel-Lindau (pVHL)-mediated ubiquitination of HIF1α, and maintains basal expression level of HIF1α, which is critical for endosome trafficking-mediated ciliogenesis [48].

## 7. Summary and Future Research Directions

Considerable progress has been made in the determination of the functions of ubiquitination in flagellar disassembly, flagellar signal transduction, spermatogenesis, and ciliogenesis. Ubiquitination is a conserved form of PTM that associates with both motile and primary cilia. However, several questions regarding ciliary/flagellar ubiquitination still need to be addressed: First, whether the ubiquitin conjugation system also presents in primary cilia, right now we only have the support evidence from the proteomic analysis [31]. Second, what are the substrates of ciliary ubiquitination when cilia undergo assembly, disassembly and how a variety of mono ubiquitin or poly ubiquitin chains are added to each substrate? In addition, the E1, E2, and substrate specific E3 need to be identified for each substrate. Third, what is the final fate of ciliary ubiquitinated proteins, reutilized or degraded in proteasome of cell body? Fourth, how to determine the specific functions of protein ubiquitination in cilia/flagella taking into account the fact that flagellar E1-activating enzyme and other components of the ubiquitin conjugation system are also found in the cell body. Fifth, how the ciliary ubiquitination events are regulated, the signal is from the cilia or from the cell body. Clearly, several ciliary research models and new technologies need to be combined to explore these questions. Addressing these gaps will further facilitate our understanding of the function of ubiquitination in cilia/flagella.
